# Loss of olfactory sensitivity is an early and reliable marker for COVID-19

**DOI:** 10.1093/chemse/bjac022

**Published:** 2022-09-07

**Authors:** Behzad Iravani, Artin Arshamian, Johan N Lundström

**Affiliations:** Department of Clinical Neuroscience, Karolinska Institutet, Stockholm, Sweden; Department of Clinical Neuroscience, Karolinska Institutet, Stockholm, Sweden; Department of Psychology, Stockholm University, Stockholm, Sweden; Department of Clinical Neuroscience, Karolinska Institutet, Stockholm, Sweden; Stockholm University Brain Imaging Center, Stockholm University, Stockholm, Sweden; Monell Chemical Senses Center, Philadelphia, Pennsylvania, United States

**Keywords:** olfactory loss, COVID-19, odor intensity, home testing

## Abstract

Detection of early and reliable symptoms is important in relation to limiting the spread of an infectious disease. For COVID-19, the most specific symptom is either losing or experiencing reduced olfactory functions. Anecdotal evidence suggests that olfactory dysfunction is also one of the earlier symptoms of COVID-19, but objective measures supporting this notion are currently missing. To determine whether olfactory loss is an early sign of COVID-19, we assessed available longitudinal data from a web-based interface enabling individuals to test their sense of smell by rating the intensity of selected household odors. Individuals continuously used the interface to assess their olfactory functions and at each login, in addition to odor ratings, recorded their symptoms and results from potential COVID-19 test. A total of 205 COVID-19-positive individuals and 156 pseudo-randomly matched control individuals lacking positive test provided longitudinal data which enabled us to assess olfactory functions in relation to their test result date. We found that odor intensity ratings started to decline in the COVID-19 group as early as 6 days prior to the test result date (±1.4 days). Symptoms, such as sore throat, aches, and runny nose appear around the same point in time; however, with a lower predictability of a COVID-19 diagnosis. Our results suggest that olfactory sensitivity loss is an early symptom but does not appear before other related COVID-19 symptoms. Olfactory loss is, however, more predictive of a COVID-19 diagnosis than other early symptoms.

Olfactory dysfunction is a key symptom of the COVID-19 disease and symptom tracking studies have demonstrated that a sudden loss of olfactory functions is the most reliable symptom of the disease (Menni et al. 2020; Gerkin et al. 2021). Anecdotal evidence indicates that olfactory loss is an early symptom appearing before other symptoms but objective measures supporting this statement are currently missing.

The key to an individual’s attempt to limit the spread of any contagious disease is monitoring early disease symptoms. At the onset of the COVID-19 pandemic, fever and cough were reported as reliable early symptoms in non-hospitalized cases and considerable monitoring effort was globally focused on these 2 symptoms (Hu et al. 2020; Wei et al. 2020). However, olfactory dysfunction soon emerged as a symptom of interest (Tostmann et al. 2020) and we now know that a great portion of individuals with confirmed COVID-19 infection report either complete or partial loss of olfactory functions (Hannum et al. 2020; Gerkin et al. 2021). Given that a large portion of all individuals with COVID-19 lose either all or some olfactory functions at some stage of the disease, it is not surprising that a reduced sense of smell is the symptom with the highest odds ratio in non-hospitalized cases (Menni et al. 2020; Rudberg et al. 2020; Gerkin et al. 2021). Olfactory loss at some stages of the disease is so prevalent that loss of olfactory functions can be used to monitor the increase of COVID-19 prevalence in a geographical area (Iravani et al. 2020; Pierron et al. 2020). In a non-clinical healthy population, the relationship between self-assessed and psychometrically assessed olfactory function is, however, poor (Landis et al. 2003). While most people will notice a sudden and complete loss of olfactory function, awareness of a partial olfactory loss is far lower than a perceptual loss in other sensory modalities, such as audition and vision. To reliably estimate olfactory loss, probing olfactory functions with actual odors is therefore needed.

At the onset of the pandemic, an international group of chemosensory scientists provided an online tool that enabled individuals to assess their olfactory performance using 5 selected common household odors from a list of 71 suggestions (Iravani et al. 2020; Snitz et al. 2022). Although the tool is anonymous to protect user privacy, individuals can continuously monitor their odor performance over time using a login mechanism. Importantly, at each login, the user completes a COVID-19 symptom check, reporting potential symptoms, such as cough, fever, etc., as well as reporting any formal COVID-19 testing they had undergone as well as the outcome of the test. Several hundred individuals used the tool to continuously assess their olfactory functions and report their potential COVID-related symptoms. Some of them contracted COVID-19 during their use of the tool, thereby creating a natural longitudinal experiment and data that enables direct comparisons between onset of olfactory dysfunction, COVID-related symptoms, and potential COVID-19 diagnosis. To this end, we used data obtained between April 2020 and February 2021, originating mainly from the Swedish first and second waves where individuals were assumably infected with one of the prevalent SARS-CoV-2 virus strains in the general Swedish population at the time in question; the wild-type, the B.1.1.7 (Alpha), and to a lesser extent, the B1.351 (Beta) variants (Public Health Agency of Sweden 2022).

Utilizing this unique longitudinal sensory data, we assessed the hypotheses that a decline in olfactory functions occurs before other COVID-related symptoms are reported by participants. Confirmation of this hypothesis would suggest that a decline in olfactory function is not only an early symptom of COVID-19 but also a symptom that occurs before other common COVID-related symptoms.

## Materials and methods

### Participants

**Fig. 1.**
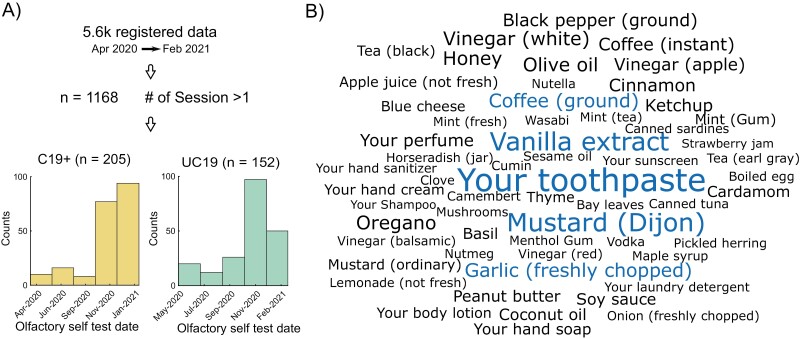
Data, self-test date density, and odors. (A) Total number of individuals and the date range of data registration from which we included only individuals with more than one session of data registration. We pseudo-randomly sampled from the undetermined COVID-19 status (UC19) cohort to match the population distribution of positive COVID-19 status (C19+) cohort. (B) The word cloud shows the odor names that are rated and highlights the more frequently rated ones in a bigger and blue font.

A total of 5,608 unique individuals enrolled, identified themselves as residing in Sweden, and entered complete data on the web-based data registry platform smelltracker.org during the 10 months between April 2020 and February 2021. We are here only assessing individuals from Sweden because our ethical permit for this assessment only covers Swedish residents and the time between the COVID test and result distribution is uniform. Moreover, because we were only interested in assessing individuals who provided longitudinal odor data, we excluded all individuals who only completed one session, as well as 161 individuals who rated all odors consistently above 95 on a 0–100 scale, leaving a total of 1,168 individuals. All the remaining individuals were above 18 years old, and their COVID-19 status was either confirmed with a PCR test, so-called C19+ (*n* = 205, 149 women and age: 43 ± 13) or not determined and labeled undetermined COVID-19 (UC19). Given that the testing date distribution of UC19 cohort was different from that of the C19+, we pseudo-randomly selected individuals from UC19 (*n* = 152, 113 women and age: 45 ± 14) to comparably match the number of individuals in 2 cohorts for a given month (Fig. 1A). The study was approved by the Swedish Institutional Review Board (Etikprövningsnämnden) and participants did not receive any form of monetary compensation for their participation and consent was waived. All aspects of the study complied with the Declaration of Helsinki for Medical Research involving human subjects.

### Procedure and data collection

All data collection was carried out via the Swedish version of the web-platform by which participants were able to create account to provide details regarding age, sex (Woman/Man/Other), and their COVID-19 test status (i.e. not tested, tested negative, tested positive). Particularly, regarding the COVID-19 status, if the participant provided no answer or marked “not tested,” we labeled them as “undetermined” (UC19). Of note, we did not include participants who marked “tested negative” in the analysis to remove bias from our results due to the notion that these individuals got tested by experiencing symptoms that were not COVID-19-related. For repeated measurement, the web-platform allows individuals to repeatedly report their COVID-19 test status as well as self-test their odor performance. Specifically, for the odor performance test, participants chose 5 household odors from a list of 71 common household items. We had participants rate 5 odors to strike a balance between increased reliability, where more assessments render more reliable data (Kern et al. 2015), and a low burden for participants to facilitate broad participation. The most frequently selected odors are illustrated in Fig. 1B. At repeated testing, the same 5 odors, freshly prepared, were used. Participants then proceeded to smell each odor and, on a separate page for each odor, rated their perceived intensity and pleasantness on visual analog scales, ranging from very weak/very unpleasant to very strong/very pleasant, respectively. These scales were coded as ranging from 0 (min) to 100 (max). Participants could smell the odors as often as they liked and there was no time pressure applied. We are here only focusing on odor intensity ratings. Moreover, in each session, participants were asked to report any experienced COVID-19 symptoms from a list of symptoms containing the following options: No symptoms, Fever, Cough, Shortness of breath or Difficulty Breathing, Tiredness, Aches, Runny nose, and Sore throat.

### Data reduction and statistical analysis

We analyzed the C19+ odor intensity ratings to determine the time-course of the potential odor intensity impairment with respect to the COVID-19 test result date. The interval during which the odor ratings were evaluated included a range between −25 and +25 days with the date of reported COVID-19 test result as day 0. This interval was logarithmically segmented into 13 bins and ratings entered during each bin were averaged. We used logarithmic bins for 2 reasons. The number of individuals exponentially decreased as we deviated from the COVID-19 test result day; using logarithmic bins prevented skewing of results due to differences in sample size within each bin. Moreover, assessing olfactory intensity ratings over a long time both before and after the COVID-19 test result day naturally leads to statistical testing of multiple time points. Using logarithmic bins allowed us to limit the number of statistical tests yet focus our statistical testing power around the date of the reported COVID-19 test result (day 0). Naturally, the number of individuals for each bin varies depending on the availability of the data for that specific bin with the maximum number of individuals occurring in the bin that includes the test result date (i.e. equal to 205, the total number of individuals in C19+). To correct for the unbalanced distribution, we used Welch’s *t*-test wherein the inequality of variances is not a concern. Moreover, we created normative baseline values of intensity within the C19+ cohort by averaging ratings 60 days before or after the test result. Because frequentist approaches are more affected by included sample size and number of tests performed, we also assessed the time-course of the intensity judgments over time within a Bayesian framework where we considered an uninformed prior for the variance on 2 levels (i.e. within and across days to account for unequal variance in a conservative manner). A half-Cauchy with a scale factor of 10 was considered to explain the inter-days variance and further a uniformed prior normal distribution with mean of 12 and standard deviation of 4 was taken into account for explaining the intra-days variance. Therefore, our new complementary Bayesian statistical model was defined as follows for each day:

Odor Intensity ~ N (mu, sigma); mu = b0 + b1 × [C19_Interval/Baseline]; sigma ~ Half-Cauchy (scale factor = 10); b1 ~ N (0, sigma_b1); sigma_b1 ~ N(12,4).

Finally, we assessed each of the COVID-19 symptoms’ time-course as a function of days with respect to the test result date. Identical data reduction was applied as described above for time-course assessment of odor intensity impairment. However, the interval for this assessment was reduced to −10 to +10 days, using the date of reported COVID-19 test result as day 0, to achieve comparable statistical power. To assess differences between symptoms, we first estimated the null occurrence probability of each symptom in the UC19 cohort. Next, using a two-sided binomial test, we determined the corresponding *z*-value for each bin within the C19+ cohort. Significant and high *z*-values, for each day, indicate that the prevalence of this specific symptom is exclusive to COVID-19 whereas significant and low *z*-values denote that this specific symptom is not exclusive to COVID-19. Finally, we followed up on this analysis using logistic regression to assess the earliest day that each specific COVID-19 symptom was manifest in relation to the test result date and if that given symptom was able to dissociate C19 from UC19. For each COVID-19 symptom, including odor intensity ratings, we fitted a logistic regression and compared the sensitivity, specificity, and the balanced accuracy, which is defined as the average of sensitivity and specificity. Nineteen unique individuals (age = 43 ± 11, 18 women) who were diagnosed with COVID-19 fulfilled the criteria for this analysis with enough longitudinal data. Consequently, we picked 21 random individuals (age = 47 ± 14, 17 women) from the UC19 cohort who registered data around the same day from a hypothetical test result day, here determined as the median of the reported test result dates (i.e. 5 December 2020). Next, for each COVID-19 symptom, including odor intensity ratings, we used the fitted logistic regression model and determined the confusion matrix as well as the balanced accuracy for predicting C19+.

## Results

### Onset of reduced odor intensity perception might occur before positive COVID-19 test

**Fig. 2.**
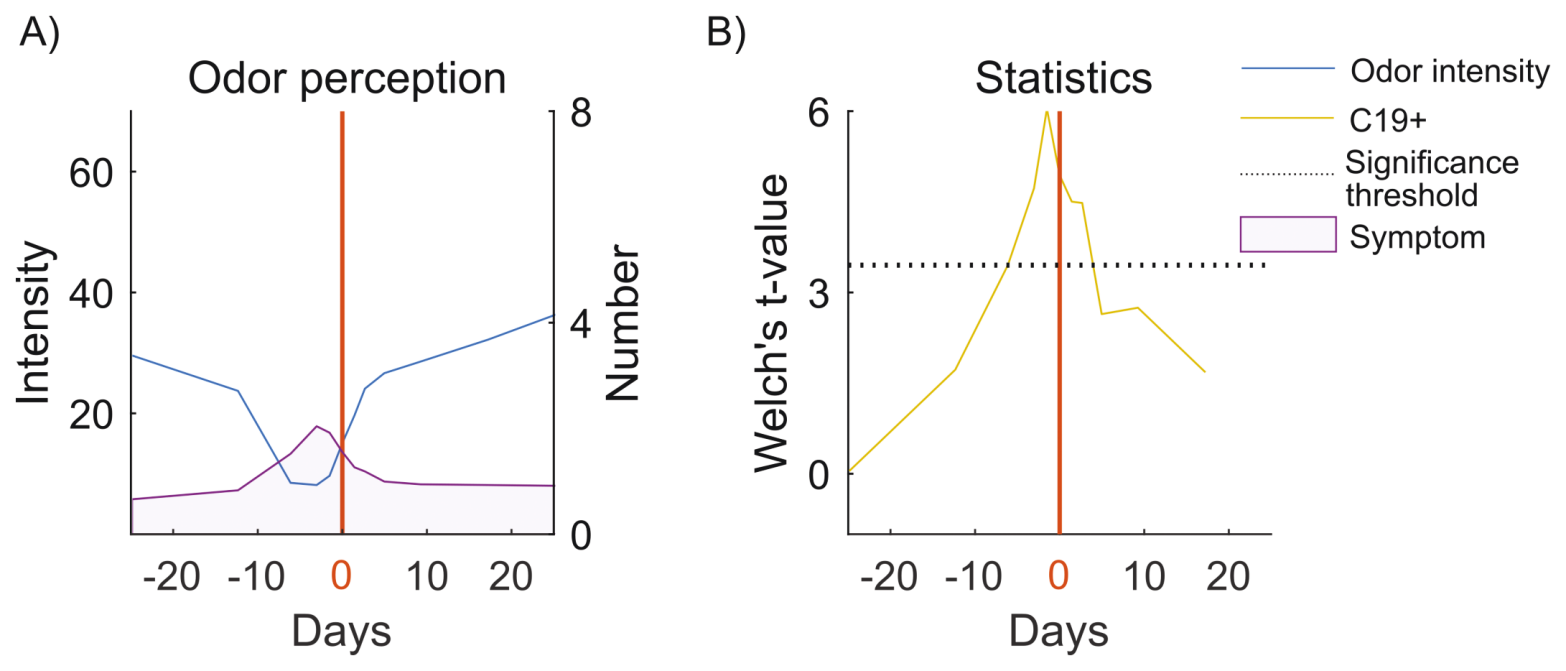
Odor intensity impairment and COVID-19. (A) The blue line indicates the median odor intensity rating as a function of days in relation to test result day (i.e. 0 and denoted with solid red line). Likewise, the purple area together with the right axis show aggregated a number of symptoms, as a function of days, locked to test result day. (B) The yellow line indicates the Welch’s *t*-value for the odor intensity measure as a function of days in relation to test result day. The significance threshold (*P* < 0.05) is shown with dotted black line.

We first sought to know whether measures of odor intensity had decreased before the individual underwent a test for COVID-19. At the time of the study, the result after returned PCR test arrived on average across the region within 2 days (Almgren and Björk 2021). To this end, we assessed the intensity ratings for C19+ across 25 days before and after the COVID-19 test result day to determine the curve of odor intensity impairment in COVID-19 over that extended time. Specifically, ratings of the 5 odors were averaged and time-locked to COVID-19 test result day. We found that the median of the odor intensity ratings started to decline in the C19+ group as early as 6 days (±1.4) prior to the test result date (i.e. denoted by 0 in Fig. 2A and B) compared to C19+ baseline (Fig. 2B), *t*(28.80) = 3.455545, *P* = 0.0014, *p*_FDR_ corrected = 0.0026, CI = [12.478, 47.886]. Moreover, we could replicate this finding using a Bayesian statistical model where we found BF_10_ = 3.17 for the day bin [−7.4, −4.6] which corresponds with our original finding at −6 days (±1.4).

### Onset of odor intensity impairment aligns with the earliest COVID-19 symptoms

**Fig. 3.**
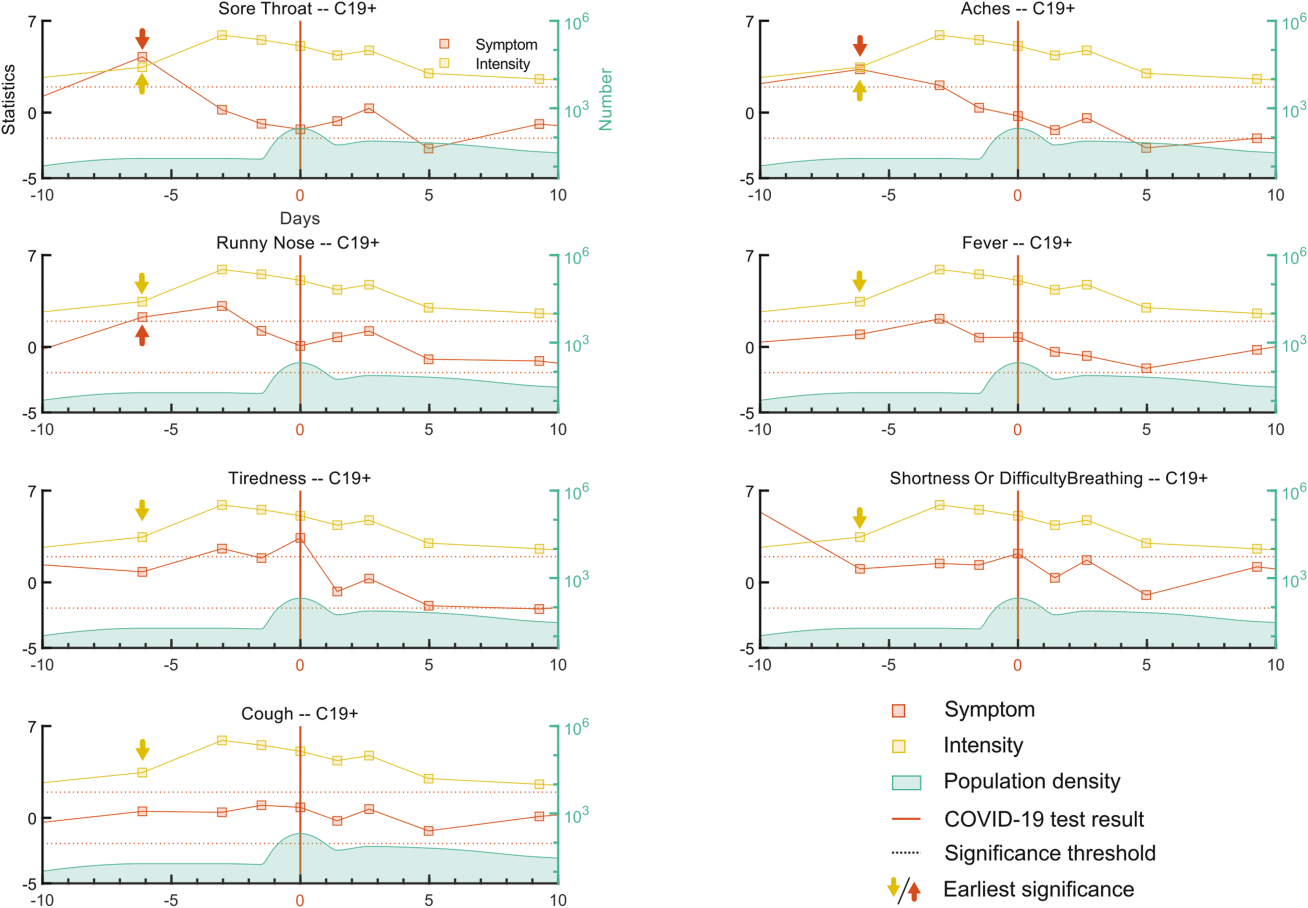
COVID-19 symptoms compared to intensity impairment time-courses. The time-course of 6 major COVID-19 symptoms, including Sore throat, Aches, Runny nose, Fever, Tiredness, Shortness of breath or Difficulty Breathing, and Cough time-courses, denoted by filled red connected squares were assessed and compared to the time-course of rated odor intensity, denoted by filled yellow connected squares. The vertical red line at 0 depicts the test result day. The green distribution together with the green axis on the right side of the plots shows the number of individuals for each specific day. Two dotted horizontal black lines show the significance threshold level. Red and yellow arrows show the earliest significant day for symptom and odor intensity inflections, respectively.

Odor intensity ratings declined as early as 6 days (±1.4) prior to the reported test result, thereby suggesting that a decline in odor intensity perception is an early sign of COVID-19. We therefore assessed whether odor intensity values were aligned with other COVID-19 symptoms. There was a significant negative association, *r*(14) = −0.95, *P* < 0.001, between median odor intensity ratings and a number of COVID-19 symptoms for C19+ over time, as determined by a Spearman rank correlation. This finding demonstrates that odor intensity impairment aligned with COVID-19 symptom progression. Next, we asked whether the onset of decline in odor intensity perception occurred earlier than other non-odor-related COVID-19 symptoms. To test this hypothesis, we first determined whether a COVID-19 symptom is significantly discernible on a specific day in the course of the disease by estimating the probability of reporting a specific symptom in the UC19 cohort. We found that probabilities of reporting COVID-19 symptoms in the UC19 group were as following (in descending order): Runny nose, *P* = 0.32; Tiredness, *P* = 0.31; Cough, *P* = 0.21; Aches, *P* = 0.12; Sore Throat, *P* = 0.11; Fever, *P* = 0.06; Shortness of Breath or Difficulty Breathing, *P* = 0.04. We considered these probabilities as our null hypothesis. Next, we assessed when, across the time-course of ratings, each COVID-19 symptom in the C19+ group significantly stood out from the baseline (i.e. the null probabilities derived from the UC19 cohort) as a function of days locked to the test result date. In other words, when each symptom might serve as an indication of COVID-19. We assessed this using two-way binomial tests separately for each symptom (Fig. 3). We found that, in addition to olfactory intensity impairment that start to differentiate the groups 6 (±1.4) days prior to the test result date, Sore throat (*z* = 4.25, *P* < 0.01), Aches (*z* = 3.30, *P* < 0.01), and Runny nose (*z* = 2.27, *P* < 0.03), are the earliest symptoms. It is worth mentioning that although the effect size for Runny nose is smaller than most of the aforementioned symptoms, it consistently stays above the significance level for 3–4 days (day −3: *z* = 3.12, *P* < 0.01). One other significant COVID-19 symptom that surpassed the significance level was Fever (*z* = 2.15, *P* < 0.05) but at a slightly later time point compared to other symptoms, around −3 days in respect to test result day (Fig. 3). It is worth noting that Shortness of Breath or Difficulty Breathing did appear to increase earlier than −6 days (±1.4), yet due to a low number of observations at the early sessions, we were not able to statistically test the symptoms probability for a wider range of days.

**Fig. 4.**
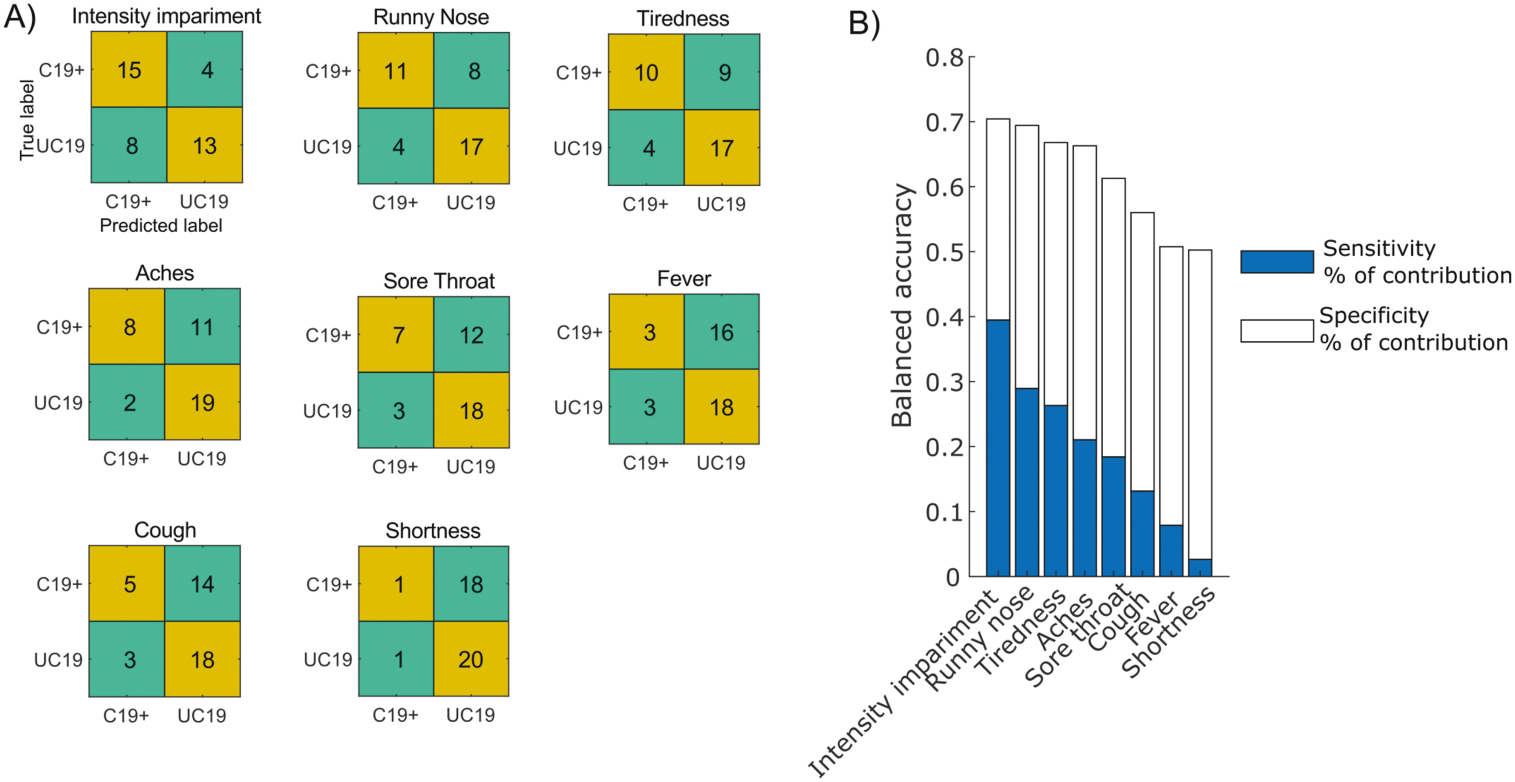
Logistic regression model of COVID-19 symptoms. (A) Confusion matrices for the different COVID-19 symptoms. The vertical axis is the true label whereas the horizontal axis is the predicted label by the logistic model. The confusion matrixes are sorted according to their performance. (B) Bars show the balanced accuracy, estimated as the percentage of contribution of sensitivity and specificity to each model. The lower and the upper segments of each bar represent the sensitivity and specificity, respectively, that contribute to the balanced accuracy.

Finally, we sought to determine which symptom in our data best predicted a COVID-19 diagnosis on the −6 days using logistic regression models fitted to the data of each symptom across 2 cohorts. In order to assess the predictive performance of our models, we calculated a confusion matrix that displays and summarize the model performance according to the known (True label) and predicted outcomes (Predicted label). To this end, the confusion matrix for each symptom’s logistic model was computed to estimate the sensitivity and specificity of that symptom. We found that odor intensity impairment has the highest balanced accuracy of 70% followed by Runny nose with a balanced accuracy of 69%. Using chi-squared test, we further found that odor intensity impairment, χ^2^ = 13.1, *P* < 0.01, Runny nose, χ^2^ = 6.61, *P* < 0.01, Aches, χ^2^ = 5.91, *P* < 0.02, and Tiredness χ^2^ = 5.06, *P* < 0.03, logistic models significantly outperformed the constant null model (Fig. 4A). The logistic model for Sore throat performed marginally better, χ^2^ = 2.75, *P* < 0.10, than the constant null model. Other symptoms’ logistic models (all *P*s > 0.34) were not significantly different from Fig. 4B).

## Discussion

We can here demonstrate that although reduced olfactory abilities are an early sign of COVID-19, it is not appearing earlier in the disease progression than several other symptoms of COVID-19. However, olfactory dysfunction was a symptom that demonstrated the highest predictability of COVID-19; a finding that has been demonstrated in several other studies using subjective measures.

Olfactory ratings started to clearly decline 6 days before participants indicated a positive test result. It is worth highlighting that all participants were regular data providers before their positive test result meaning that we were in a unique position to assess odor ratings before a potential test result might bias their ratings. It is not possible to definitely know, however, at what point in time participants were infected because test results might be communicated with different delays and participants did not provide information how much time had elapsed from test result and first post-result data entry. Nonetheless, it is clear that olfactory dysfunction was not only a common COVID symptom but also an early one, appearing around 4 days prior to testing for COVID-19. Although it was a more reliable symptom during the early occurring strains of the SARS-CoV-2 virus (Rudberg et al. 2020), olfactory loss did not, however, seem to occur earlier than other common signs of COVID-19.

The main pathway for the SARS-CoV-2 virus into the body is thought to be the angiotensin-converting enzyme 2 (ACE2) receptor; a receptor that is expressed throughout the human respiratory system with high density in the nasal epithelium and especially in the supporting sustentacular cells (Fodoulian et al. 2020; Hou et al. 2020; Muus et al. 2021). It is therefore not surprising that reduced olfactory function is an early sign of COVID-19, appearing already around 6 days before participants reported their positive test. It is not possible to exactly know when in time, in relation to their reported positivity, participants were infected. However, it can be assumed that all participants using the webpage were familiar with media reports of the link between olfactory loss and COVID-19 and therefore can be assumed to have an interest to quickly perform their next olfactory assessment after receiving news of positive COVID-19 tests. Given that the average incubation time of the SARS-CoV-2 virus being reported as 5–6 days (McAloon et al. 2020), the decline in olfactory sensitivity can then be assumed to occur within the first days after infection.

Because the time of testing of included participants stretches over almost a year, several strains (Variants Being Monitored [VBMs]) of the SARS-CoV-2 can be assumed to have infected included participants. It is not possible to know exactly what proportions of VBMs dominated in our sample and when but the wild-type strain, the B.1.1.7 (Alpha), and to a lesser extent, the B1.351 (Beta), were the dominating VBMs in the general Swedish population at the time in question (Public Health Agency of Sweden 2022). Whether olfactory loss is an early and reliable sign of COVID-19 infection also for the B.1.1.529 and BA.1-2 (variants of Omicron) VBMs is, at the time of submission in May 2022, still debated. Tentative data originating from the verbal track-and-trace program in the United Kingdom suggests, however, that fewer individuals report subjective olfactory dysfunction after infection with the Omicron variant (Vihta et al. 2022). That said, these subjective data are collected already 1–2 days after a positive test and it is not yet determined whether potential lower numbers are due to a delay in onset of olfactory dysfunction or whether reports that the Omicron variant, in contrast to previous VBMs, often causes a nasal discharge or congestion might affect these early results (Vihta et al. 2022).

In the present study, we assess olfactory functions using intensity ratings of common household odors. In most studies on COVID-19 influence on the olfactory system, olfactory function has either not been assessed, assessed using subjective self-reports, or assessed with cued olfactory identification performance. While most people do notice a sudden and complete loss of olfactory function, awareness of a partial loss is far lower than a comparable perceptual loss in other sensory modalities like audition and vision (Landis et al. 2003). Cued identification performance alone is a crude measure of olfactory function that is most suitable to detect anosmia given the use of strong odors, that difficulty level is partly decided by the similarity between the presented odors and the lures on the cue card, and its partial reliance on cognitive and language skills (Larsson et al. 2004; Hedner et al. 2010). Therefore, to reliably estimate olfactory loss that does not border on anosmia, it is beneficial to probe aspects of olfactory function that are linked to the individual’s sensitivity. Odor intensity estimates are linked to the individual’s odor detection threshold (Cain 1969). However, of higher relevance here is degree of fluctuation over time and based partly on the same data, we previously estimated the test–retest reliability of online odor intensity measure as 0.66, a value nearly identical to another study assessing test–retest of odor intensities (Kern et al. 2015). Moreover, this value is in-line with common odor detection thresholds where reliability between 4 time points has been reported in the range of 0.43–0.85 (Albrecht et al. 2008).

In conclusion, we can demonstrate that for individuals infected by the SARS-CoV-2 virus, odor intensity ratings start to decline as early as 6 days prior to their reported test result. However, other symptoms of COVID-19, such as aches, shortness of breath, and sore throat appear around the same point in time. These non-olfactory-related symptoms display lower predictability of a COVID-19 diagnosis. Our results demonstrate that olfactory dysfunction is an early symptom of COVID-19 but not a symptom that appears before other related COVID-19 symptoms.
